# Patient Recruitment Challenges in Academic Clinical Research: Perceptions and Experiences of Healthcare Professionals in a Teaching Hospital

**DOI:** 10.7759/cureus.102601

**Published:** 2026-01-29

**Authors:** Vimala Ananthy, RP Priyadharsini, Arunkumar Muthalu, Umamaheswari Subramanian

**Affiliations:** 1 Pharmacology, Mahatma Gandhi Medical College and Research Institute, Sri Balaji Vidyapeeth (Deemed to be University), Puducherry, IND; 2 Pharmacology, Jawaharlal Institute of Postgraduate Medical Education and Research (JIPMER), Karaikal, IND; 3 Critical Care Medicine, Mahatma Gandhi Medical College and Research Institute, Sri Balaji Vidyapeeth (Deemed to be University), Puducherry, IND; 4 Pharmacology, Employees' State Insurance Corporation (ESIC) Medical College & Hospital, Chennai, IND

**Keywords:** academic hospital, barriers, clinical research, patient recruitment, questionnaire survey

## Abstract

Background: Academic clinical studies often struggle most with recruiting patients, and poor recruitment can delay projects and weaken the quality of study findings. Because recruitment in teaching hospitals is usually led by residents, faculty, and nurses, understanding what they experience on the ground is essential to improve enrolment in academic settings. This study explores the practical factors that facilitate or hinder patient enrolment as perceived by healthcare providers working in a tertiary care teaching hospital.

Methods: We conducted a cross-sectional, questionnaire-based study among 120 healthcare professionals (40 residents, 40 faculty, and 40 nurses) in a tertiary care teaching hospital. Participants provided anonymous responses using a validated 10-item Likert scale instrument covering demographics and perceived recruitment barriers. Data were analyzed using descriptive statistics, along with Chi-squared tests and ANOVA to compare differences across groups. Open-ended responses were analyzed using thematic coding.

Results: The most commonly reported barriers were time constraints (99, 82.5%), difficulty explaining trials to patients (86, 71.6%), restrictive eligibility criteria (76, 63.3%), and administrative workload (43, 58.3%). Residents reported greater difficulty related to limited time during clinical duties, while faculty more often highlighted the burden of administrative tasks. Prior research training was associated with better confidence in explaining studies (p = 0.014) and greater motivation to actively identify eligible participants (p = 0.028). Thematic analysis grouped barriers into three broad domains: operational constraints, communication challenges, and limited institutional support. Participants suggested practical solutions, including appointing research coordinators, providing multilingual consent forms, and implementing structured training programs for research teams.

Conclusion: Recruitment barriers in academic clinical research are largely driven by operational pressures and communication gaps, compounded by limited organizational support. Strengthening institutional systems-particularly through dedicated support staff and structured training-may improve recruitment efficiency. Building research competencies among residents, nurses, and faculty could promote more ethical, timely, and effective patient enrolment in academic clinical studies.

## Introduction

Clinical research often hinges on the successful recruitment of patients. When enrollment is slow, studies run behind schedule, costs rise, and sample sizes fall-ultimately reducing statistical power and the reliability of results [1]. Published estimates suggest that a large proportion of clinical studies struggle to meet recruitment targets (often quoted in the range of 60%-80%), which translates into longer timelines and higher overall expenditure [2,3].

In academic hospitals-especially in developing-country settings-recruitment becomes even more difficult because the same staff are expected to balance clinical service, teaching, and administrative responsibilities. With limited time and competing priorities, recruitment can be treated as an “extra task,” even though inadequate enrollment remains one of the most common reasons studies fail to deliver meaningful conclusions [4].

Recruitment is influenced by factors operating at multiple levels. Common barriers include lack of time, heavy outpatient workload, restrictive eligibility criteria, communication challenges, and concerns related to research participation-such as fear of trial risks, doubts about randomization, and mistrust. Successful recruitment depends not only on identifying eligible patients but also on the clinician’s ability to explain the study clearly, obtain truly informed consent, follow protocols, and work within ethical and governance frameworks [5]. When healthcare providers feel underprepared to communicate research details or address patient concerns, they may avoid recruitment conversations altogether, reducing enrollment opportunities [6].

India presents additional context-specific challenges. Hospital systems often fail to fully integrate research workflows, despite a large patient volume and a growing clinical research footprint. Cultural beliefs, language barriers, fear of exploitation, and limited awareness of clinical trial procedures may shape patient willingness to participate [7]. Many academic centers function without full-time clinical research coordinators, structured recruitment support, or streamlined processes for low-risk studies. As a result, recruitment frequently depends on busy treating clinicians who may need extra training for research documentation and communication tasks. Limited infrastructure and manpower support can create bottlenecks that affect both patient experience and study progress [8].

Importantly, much of the existing literature focuses on principal investigators and senior faculty, while the contributions and difficulties of residents and nursing staff-who often interact with patients first-remain underexplored. In reality, recruitment is a shared workflow: residents manage day-to-day clinical care, nurses provide counseling and continuity, and faculty guide treatment decisions and research oversight [9-11]. Understanding recruitment requires studying these groups together as a linked system. Evidence from Western settings may not translate directly to Indian academic hospitals because of differences in workload patterns, ethics pathways, staffing models, and patient expectations. There is a need for locally relevant, feasible, ethical, and cost-effective solutions tailored to busy tertiary care environments.

This study, therefore, examines healthcare professionals’ perspectives on patient enrollment for academic clinical research in a teaching hospital. By combining Likert-scale ratings with open-ended responses, it aims to quantify key operational barriers, explore underlying causes, and identify realistic strategies to improve recruitment-such as multilingual patient materials, structured research training, simplified consent workflows, and better institutional support-without adding undue burden to clinical staff. Strengthening recruitment processes can improve study success, enhance ethical standards, shorten research timelines, and ultimately support stronger evidence-based practice.

## Materials and methods

Study design and setting

We used a quantitative, cross-sectional survey design to understand the difficulties healthcare professionals face while enrolling patients in academic clinical studies. The research was carried out in a 1,400-bed tertiary care teaching hospital operating under a deemed university model (Mahatma Gandhi Medical College and Research Institute, Sri Balaji Vidyapeeth (Deemed to be University), Puducherry), integrating clinical services with academic research. Data were gathered over a one-month period (October 2025) while standard medical, surgical, and outpatient services were maintained. Surveys were administered during regular working hours to capture experiences reflective of real-world recruitment conditions.

As this was an exploratory single-center survey, no formal power calculation was undertaken; instead, sampling was feasibility-based within the one-month study period and guided by the need for balanced representation across residents, faculty, and nurses. We also noted the Institutional Ethics Committee (IEC)-approved upper limit (n = 150) and that 120 complete responses (40/group) were analyzed to enable group comparisons and stable estimation of Likert mean scores. This distribution allowed comparison across groups while remaining feasible within institutional resource constraints. Survey links and forms were shared via departmental announcements and direct invitations. The study included three groups of healthcare professionals who are routinely involved in patient care and clinical research activities: medical residents who manage day-to-day clinical work along with research duties, faculty members who oversee protocols and coordinate enrollment processes, and nursing staff who support patient counseling, consent procedures, and follow-up. Participants had to have at least six months of clinical experience, be directly involved in patient care, and agree to take part in the study. We excluded administrative staff, interns, and personnel engaged only in indirect care or purely administrative research tasks, as their recruitment exposure differed and could bias responses.

Study instrument

We developed a structured questionnaire, which was designed to capture both the background of participants and their real-world experiences with patient recruitment, as provided in the appendix. The items were drafted after reviewing common recruiter-reported barriers in clinical research literature [1,4,9]. The tool was original and did not reproduce any copyrighted scale items. It first collected basic demographic and professional details such as age, gender, role, department, and prior research exposure. This was followed by a 10-item Likert scale (ranging from 1 = strongly disagree to 5 = strongly agree) that asked participants to rate how strongly they experienced common recruitment barriers, including lack of time, communication difficulties, ethical and language concerns, restrictive eligibility criteria, training gaps, and limited institutional support. To ensure we also captured practical insights beyond fixed-response items, the questionnaire included two open-ended questions where participants could describe the key obstacles they face and suggest ways the institution could improve recruitment. The survey was provided in English and made available both as paper copies and as a secure Google Forms link (Google, Mountain View, CA, US), so staff could complete it in a way that suited their work schedules. To ensure quality and credibility, three subject experts from pharmacology, internal medicine, and nursing validated the tool's content, resulting in a strong content validity index (CVI) of 0.87. It was then pilot-tested with 10 healthcare professionals outside the final study sample to assess clarity and completion time, leading to minor refinements. Finally, internal consistency was confirmed with a Cronbach’s alpha of 0.82, indicating satisfactory reliability.

Before recruiting, the IEC gave its approval (MGMCRI/2025/04/IEC/121). All participants received an information sheet explaining the study purpose, voluntary participation, confidentiality measures, and estimated completion time (10-15 minutes). Written informed consent was obtained before participation.

Data collection and confidentiality

Data were collected using both paper-based questionnaires and digital forms distributed through email and WhatsApp (Meta Platforms, Inc., Menlo Park, CA, US) groups. Participants completed the survey during breaks or during non-clinical intervals. Responses were anonymous, and no identifying information (name, staff ID, or contact details) was collected.

Paper responses were entered into Microsoft Excel (Microsoft Corp., Redmond, WA, US) by two independent data entry personnel to minimize errors; digital submissions were exported directly. A final verification step was performed to detect discrepancies. Data were stored on a password-protected institutional computer with access restricted to the principal investigator.

Statistical and qualitative analysis

We collected data using a specially designed, structured questionnaire that aimed to be both simple to complete and detailed enough to reflect real recruitment challenges. It began with basic demographic and professional information such as age, gender, role, department, and previous research experience. Participants then responded to a 10-item Likert scale (from 1 = strongly disagree to 5 = strongly agree) that explored common barriers to patient enrollment, including lack of time, difficulties in communication, ethical or language-related issues, strict eligibility criteria, gaps in training, and limited institutional support. To capture richer, practical insights, the questionnaire also included two open-ended questions where participants could describe the main obstacles they face and suggest realistic improvements the institution could adopt. The survey was provided in English and was available both as paper forms and as a secure Google Forms link so that staff could respond based on what suited their work schedule. To ensure the questionnaire was sound, it was reviewed by three experts from pharmacology, internal medicine, and nursing for clarity and relevance, achieving a CVI of 0.87. It was then pilot-tested with 10 healthcare professionals outside the final study sample, and small refinements were made. Reliability was confirmed with a Cronbach’s alpha of 0.82, showing good internal consistency.

Likert responses were summarized using mean ± SD for interpretability and compared across groups using one-way ANOVA. While Likert-scale data are ordinal, ANOVA is considered reasonably robust for 5-point items with balanced group sizes and similar variances; therefore, we used it as an approximate method for detecting between-group differences in perceived barrier intensity. Open-ended responses were analyzed using an inductive thematic approach. Two investigators independently reviewed all responses to become familiar with the data and generated initial codes line-by-line. A preliminary codebook was developed through discussion and applied to the full dataset. Codes were then grouped into higher-order categories and refined into themes by iterative comparison across participant groups (residents, faculty, and nurses). Discrepancies in coding were discussed and resolved by consensus; when consensus was not reached, a third investigator adjudicated. Themes were finalized when no new concepts emerged, and representative, anonymized excerpts were selected to support each theme.

## Results

Demographic and professional characteristics

Overall, 34 (28.3%) had more than five years of experience. Among faculty members, 21 (52.5%) reported prior involvement as clinical trial investigators or coordinators (Table 1).

**Table 1:** Demographic profile of study participants (N = 120)

Variable	Residents (n = 40)	Faculty (n = 40)	Nurses (n = 40)	Total (N = 120)
Mean age (years), mean ± SD	28.4 ± 2.7	38.5 ± 5.2	29.0 ± 3.6	31.8 ± 6.4
Female, n (%)	21 (52.5%)	19 (47.5%)	30 (75.0%)	70 (58.3%)
Clinical experience > 5 years, n (%)	0 (0.0%)	24 (60.0%)	10 (25.0%)	34 (28.3%)
Prior research training, n (%)	10 (25.0%)	21 (52.5%)	12 (30.0%)	43 (35.8%)
Past involvement in clinical trials, n (%)	12 (30.0%)	23 (57.5%)	9 (22.5%)	44 (36.7%)

The research participants named their patient recruitment barriers as their busy schedules, their difficulties with explaining study protocols, and their rigid enrollment criteria. The residents encountered the most difficulties because their work schedule did not provide enough time (4.4 ± 0.8), although faculty members faced additional administrative obstacles (4.1 ± 1.0) (p = 0.032) (Table 2). The participants reported that administrative procedures either took too long to complete or were difficult to handle at a rate of 70 (58.3%). The research findings demonstrated that participants who received research training beforehand explained study information to patients better than untrained participants (2.8 ± 1.0 vs. 3.6 ± 0.9) (p = 0.014), and they demonstrated stronger recruitment participation motivation (p = 0.048).

**Table 2:** Mean Likert scale scores for perceived barriers across professional groups *p < 0.05 is considered statistically significant. Subgroup analysis of perceived recruitment barriers using mean Likert scores across residents, faculty, and nurses. p-values were calculated using one-way ANOVA.

Barrier (score range 1–5)	Residents mean ± SD	Faculty mean ± SD	Nurses mean ± SD	p-value
Lack of time due to clinical workload	4.4 ± 0.8	4.0 ± 0.9	3.7 ± 1.0	0.021*
Difficulty explaining study details	3.7 ± 0.9	3.1 ± 1.0	3.3 ± 0.8	0.036*
Administrative burden	3.9 ± 0.9	4.1 ± 1.0	3.4 ± 0.8	0.032*
Language barriers	3.2 ± 1.1	3.0 ± 1.1	3.4 ± 1.0	0.281
Restrictive eligibility criteria	3.8 ± 0.9	3.6 ± 1.0	3.9 ± 0.7	0.412
Lack of research training	3.9 ± 0.8	2.8 ± 1.0	3.5 ± 0.9	0.003*
Motivation to recruit	3.1 ± 1.1	3.7 ± 1.0	3.2 ± 0.9	0.048*

Among the 43 participants who had received research training earlier, the overall pattern was positive. Compared with those without prior training, they reported a better understanding of study details (mean scores 3.6 vs. 2.8; p = 0.014) and felt more motivated to actively identify and approach eligible patients for recruitment (3.8 vs. 3.1; p = 0.028). They also perceived a lower burden from administrative work (3.3 vs. 3.9; p = 0.033). Overall, prior training appeared to strengthen participants’ grasp of study procedures and increased their confidence in explaining the study to patients in a clearer and more comfortable way (Table 3).

**Table 3:** Influence of prior training on perceived recruitment challenges (N = 120) *p-value < 0.05 is considered to be statistically significant. Mean Likert scores between participants with and without prior research training demonstrate statistically significant improvements in motivation and communication ability among trained individuals.

Barrier	Trained (n = 43) mean ± SD	Not trained (n = 77) mean ± SD	p-value
Difficulty explaining study details	2.8 ± 1.0	3.6 ± 0.9	0.014*
Perception of administrative burden	3.3 ± 0.9	3.9 ± 1.0	0.033*
Lack of motivation to recruit	3.1 ± 1.0	3.8 ± 1.1	0.028*
Perception that patients are unwilling	3.0 ± 0.8	3.5 ± 0.9	0.062
Lack of confidence in informed consent	2.6 ± 0.9	3.4 ± 1.0	0.017*

The open-ended responses pointed to three broad themes, with two standing out. First, many participants described operational constraints-the outpatient department is often running at full capacity, leaving very little time for detailed counseling and informed consent, and the recruitment process can feel unrealistic when most patients are focused on receiving routine care rather than considering study participation. Second, there were strong communication-related concerns: staff found it especially difficult to explain concepts like randomization and placebo in a way patients could understand, existing consent forms were not always suitable for people who speak different languages, and some patients became hesitant or mistrustful because they felt they were being “experimented on” rather than offered a structured and ethical research option.

The participants felt that improving recruitment in the hospital would require a few practical, system-level changes rather than expecting clinicians to “do more” on top of an already heavy workload. Many suggested appointing dedicated research coordinators to take care of documentation, follow-up, and day-to-day coordination. They also recommended short, focused research training sessions for residents and nursing staff, since these groups often initiate patient conversations but may not feel fully confident explaining study procedures. Participants felt that simple visual aids-such as charts, illustrations, or short handouts-could make study information easier for patients to understand. They also made a point to streamline ethics approval for minimal-risk studies so that delays and paperwork do not discourage research teams. Finally, they felt that staff who actively contribute to recruitment should receive recognition, whether through small incentives or public appreciation, as this can improve motivation and create a supportive research culture.

## Discussion

Overall, this study explored how healthcare professionals perceive recruitment barriers in a teaching hospital-based academic research setting. The findings suggest that recruitment difficulties rarely arise from a single factor; instead, they reflect a combination of limited time, communication challenges, strict eligibility criteria, and administrative workload. A key insight from the study was that those with prior research training appeared better prepared-they reported greater ease in understanding study procedures, felt more confident explaining trials to patients, and showed higher motivation to recruit participants. This highlights the value of competency-building programs in strengthening recruitment outcomes.

Time pressure emerged as the strongest barrier, with 99 (82.5%) of participants reporting it as their main difficulty. Residents, in particular, struggled the most because of long clinical shifts, high patient volume, and the absence of protected time for research activities. In many cases, recruitment had to be squeezed into already busy outpatient and ward routines, making it difficult to conduct proper counseling and obtain informed consent. This reflects a broader reality in teaching hospitals, where clinical service demands often overshadow research responsibilities-especially in settings with large outpatient loads and limited staffing.

Communication was the next major challenge, reported by 86 (71.7%) of participants. Explaining research concepts such as randomization, placebo use, follow-up requirements, adverse event monitoring, and the right to withdraw can be difficult, particularly when patients are anxious, unwell, or unfamiliar with clinical research. Faculty members generally performed better in these discussions, likely because of their greater exposure to research and experience in patient counseling. Participants also emphasized that language barriers make communication even harder, and many felt that multilingual consent forms and structured communication training would directly improve recruitment.

Administrative burden was another important barrier, and it was most strongly felt by faculty members. Participants described delays caused by prolonged approval timelines, extensive documentation requirements, repeated modifications, and multiple layers of coordination. In many Indian academic centers, where research coordinator support may be limited or absent, clinicians end up managing large amounts of paperwork themselves, reducing the time available for patient interaction and decreasing enthusiasm for recruitment.

Strict eligibility criteria also contributed to recruitment problems. Participants noted that many studies exclude patients who reflect “real-world” hospital populations, meaning eligible participants are relatively few even in high-volume clinical settings. While narrow criteria may improve internal validity, overly restrictive entry standards can make recruitment impractical and limit how widely results can be applied. Participants felt institutions and investigators should aim for a workable balance between scientific rigor and operational feasibility.

The qualitative findings strongly supported the quantitative results. Participants consistently described outpatient departments functioning at full capacity, a lack of dedicated time for research, difficulty simplifying medical language for patients, fear and mistrust among patients about being “tested,” and limited availability of study information in regional languages. Alongside these barriers, they also offered several realistic solutions-research coordinators, patient-tracking systems, simplified consent language, visual aids, and recognition for recruitment efforts-all of which could be implemented with minimal additional cost if integrated into existing hospital systems.

The study suggests that academic institutions can improve recruitment by strengthening their research infrastructure through a few core steps: appointing clinical research coordinators, building structured training in Good Clinical Practice and informed consent communication, simplifying processes for low-risk studies, ensuring multilingual patient materials, recognizing staff contributions, and providing protected time for research-related tasks. These changes could improve recruitment without increasing burnout and while maintaining ethical standards.

Where our work adds useful nuance is in showing how training and communication confidence shape recruitment behavior within a busy teaching hospital workflow. Previous evidence suggests that many participants do not fully understand key consent elements and that improving understanding often requires better communication methods and more structured, participant-centered consent processes [5]. In parallel, recruiter-focused training interventions-particularly those that help clinicians respond to patient preferences and uncertainty more skillfully-have been reviewed as a practical way to improve recruitment conversations and reduce avoidable missed opportunities [12]. In the Indian context, qualitative evidence shows that participation decisions are strongly influenced by trust, perceived risk, awareness, and communication clarity-making our participants’ emphasis on language barriers, fear of being “tested,” and the need for simpler, multilingual materials especially consistent with what patients and communities report as meaningful determinants [7].

Beyond identifying barriers, our findings point to the need for a more “upstream” and structured approach to recruitment that begins at protocol development rather than during active enrollment. A recruitment-planning framework proposed by the Clinical Trials Transformation Initiative highlights that downstream problems (missed eligible participants, poor consent conversations, and delays) often reflect earlier gaps in feasibility assessment, site workflow mapping, and communication planning [13]. In this context, embedded recruitment-support methods such as the QuinteT Recruitment Intervention (QRI) are particularly relevant because they use focused qualitative diagnostics to identify where recruitment conversations and processes break down and then implement practical, trial-specific corrective actions [14,15]. Given that our participants repeatedly emphasized difficulty explaining randomization and consent elements, it is also notable that randomized evidence shows well-designed multimedia aids (especially those combining audio with visual reinforcement) can improve participant understanding compared with text-only materials-supporting the feasibility of using brief visual tools or narrated information sheets in busy outpatient settings [16]. Finally, approaches that build trust and reduce “cold” recruitment encounters-such as structured community engagement, partnerships with local clinicians, and designated engagement coordinators-have been shown to improve screening and enrollment in pragmatic trials and may be adaptable to tertiary care hospitals seeking to improve participation among diverse linguistic and socio-cultural groups [17].

The recruitment literature consistently reports layered barriers rather than single-point problems, which our findings closely align with. In the classic review of UK-funded trials, recruitment shortfalls were commonly linked to practical issues such as site workload, trial logistics, and the day-to-day realities of delivering care alongside research [1]. Likewise, evidence syntheses have consistently highlighted clinician-side barriers like lack of time, limited staffing support, and the perceived “extra work” of trials-issues that mirror the strong time-pressure signal in our residents and the administrative burden seen among faculty [4]. Importantly, this matters because poor recruitment is not a minor inconvenience; it is a well-recognized driver of trial discontinuation and research waste, reinforcing why even “routine” operational barriers deserve institutional attention [3].

This study has strengths, including the inclusion of residents, faculty, and nurses-three groups who contribute to recruitment at different stages of patient care-as well as the combination of quantitative and qualitative methods, which allowed both measurement and deeper explanation. However, the findings should be interpreted with some limitations in mind. Being a single-center study, the results may not capture recruitment challenges faced in smaller hospitals or rural settings. Convenience sampling and self-reported responses may introduce bias, and the study did not directly include patient perspectives, which are essential to fully understand refusal or hesitation. Future studies could expand across multiple centers, include patient interviews, and evaluate recruitment interventions over time to build stronger evidence.

## Conclusions

In this single-center cross-sectional survey, healthcare professionals reported key perceived barriers to academic clinical research recruitment, particularly time constraints, communication difficulties, administrative burden, and restrictive eligibility criteria. Prior research training was associated with higher self-reported confidence in explaining studies and greater motivation to identify eligible participants. Given the observational, self-reported design and feasibility-based sampling, these findings should be interpreted cautiously and not as causal effects. Multicenter studies incorporating patient perspectives and robust sampling methods are needed to confirm generalizability and to evaluate whether targeted training and institutional supports lead to measurable improvements in recruitment outcomes.

## Appendices

Recruitment Barriers Questionnaire (author-developed; 10 items; 5-point Likert scale)

Response options for Items 1-10: 1 = Strongly disagree, 2 = Disagree, 3 = Neutral, 4 = Agree, 5 = Strongly agree.

**Table 4:** Recruitment Barriers Questionnaire Open-ended questions: In your experience, what are the main obstacles to recruiting patients into academic clinical studies in this hospital? What practical steps should the institution take to improve recruitment?

Item	Domain	Statement
1	Workload/time	I do not have enough time during routine clinical duties to discuss research studies with patients.
2	Communication	I find it difficult to explain study procedures in a way that patients understand.
3	Communication	I find it challenging to explain randomization and placebo/blinding to patients.
4	Administration	Research-related documentation and approvals create an excessive workload for recruitment.
5	Eligibility	Eligibility criteria in academic studies are often too restrictive for real-world patients seen in our setting.
6	Patient factors	Patients are often unwilling to participate due to fear, mistrust, or misconceptions about research.
7	Language/culture	Language differences and limited multilingual materials reduce recruitment effectiveness.
8	Training	I feel insufficiently trained to approach patients and counsel them for study participation.
9	Ethics/consent confidence	I lack confidence in ensuring truly informed consent during busy clinical workflows.
10	Motivation	I find it difficult to remain motivated to actively identify and approach eligible patients for studies.
